# Changing the role of traditional birth attendants in Yirol West County, South Sudan

**DOI:** 10.1371/journal.pone.0185726

**Published:** 2017-11-02

**Authors:** Calistus Wilunda, Giovanni Dall’Oglio, Chiara Scanagatta, Giulia Segafredo, Bhekumusa Wellington Lukhele, Risa Takahashi, Giovanni Putoto, Fabio Manenti, Ana Pilar Betrán

**Affiliations:** 1 Department of Pharmacoepidemiology, Graduate School of Medicine and Public Health, Kyoto University, Kyoto, Japan; 2 Doctors with Africa CUAMM, Rumbek, South Sudan; 3 Doctors with Africa CUAMM, Padua, Italy; 4 Department of Global Health and Socio-Epidemiology, Graduate School of Medicine and School of Public Health, Kyoto University, Kyoto, Japan; 5 Department of Nursing Science, Faculty of Health Care, Tenri Health Care University, Tenri City, Nara, Japan; 6 UNDP/UNFPA/UNICEF/WHO/World Bank Special Programme of Research, Development and Research Training in Human Reproduction, Department of Reproductive Health and Research, World Health Organization, Geneva, Switzerland; National Academy of Medical Sciences, NEPAL

## Abstract

Effective from May 2014, community-based traditional birth attendants (TBAs) in Yirol West County, South Sudan, were directed to start referring all women in labour to health facilities for childbirth instead of assisting them in the villages. This study aimed to understand the degree of integration of TBAs in the health system, to reveal the factors influencing the integration, and to explore the perceived solutions to the challenges faced by TBAs. A qualitative study utilising 11 focus group discussions with TBAs, 6 focus group discussions with women, and 18 key informant interviews with members of village health committees, staff of health facilities, and staff of the County Health Department was conducted. Data were analysed using qualitative content analysis. The study found that many TBAs were referring women to health facilities for delivery, but some were still attending to deliveries at home. Facilitators of the adoption of the new role by TBAs were: acceptance of the new TBAs’ role by the community, women and TBAs, perceptions about institutional childbirth and risks of home childbirth, personal commitment and motivation by some TBAs, a good working relationship between community-based TBAs and health facility staff, availability of incentives for women at health facilities, and training of TBAs. Challenges of integrating TBAs in the health system included, among others, communication problems between TBAs and health care facilities, delays in seeking care by women, insecurity, lack of materials and supplies for TBAs, health system constraints, insufficient incentives for TBAs, long distances to health facilities and transportation problems. This study has revealed encouraging developments in TBAs’ integration in the formal health system in Yirol West. However, there is need to address the challenges faced by TBAs in assuming their new role in order to sustain the integration.

## Introduction

The era of Millennium Development Goals witnessed a 59% increase in the deliveries assisted by skilled birth attendants (SBAs) and a 44% reduction in Maternal Mortality ratio (MMR) worldwide [1, 2]. Despite this achievement, each year, 45 million women still deliver without skilled attendance [1] and 303,000 die from complications related to pregnancy or childbirth worldwide [2]. Almost all maternal deaths (99%) occur in developing countries, with sub-Saharan Africa accounting for 66% of the deaths [2]. Thus, maternal mortality remains an agenda for global development as reflected in the Sustainable Development Goals [3].

Between the 1970s and 1990s, the international response to maternal mortality included training of traditional birth attendants (TBAs) [4] to attend to deliveries. Although TBAs working in well-structured contexts may reduce perinatal deaths, stillbirths, and neonatal deaths [5], their training failed to reduce maternal mortality [6]. Thus, the use of skilled birth attendants (SBAs) became the key strategy to reduce maternal mortality in developing countries.

Nonetheless, in settings where SBAs are scarce and barriers to service access abound, TBAs still attend to a majority of childbirths [7–9]. In such contexts, the disconnection between TBAs and the formal health system may impede access to maternal health services [10]. Given that integration of TBAs into the health system may increase skilled birth attendance [11–15], there is a renewed interest in the linkage between TBAs and SBAs; with TBAs working as promoters of institutional childbirth [16].

Maternal mortality ratio (per 100,000 births) is estimated to have increased in South Sudan from 763·8 in 1990 to 956·8 in 2013 and is projected to remain in the range of 500 to 925 by 2030 [17]. This is due to a fragile health system, which has been exacerbated by decades of conflict. The provision of health services in the country is hampered by numerous challenges including a chronic shortage of professional health workers. Since 2012, Yirol West County Health Department (CHD) has been partnering with Doctors with Africa CUAMM (hereafter CUAMM), an Italian non-governmental organisation (NGO), to strengthen the delivery of primary health care services in the county. The county, however, lacks skilled health workers, especially in peripheral health facilities. To fill the gap, the Ministry of Health recruited and trained some TBAs to work in health facilities (hereafter referred to as facility-based TBAs). Most TBAs, however, continued to work in villages unsupervised (hereafter referred to as community-based TBAs).

Effective from May 2014, in line with the national guidelines aimed at improving the quality of primary health care services, the county authorities banned TBAs from attending to home births and directed that all women in labour be referred to health facilities. The community-based TBAs’ main task became referring women to health facilities for childbirth. However, the TBAs were also trained for three days on assessing pregnant women, detecting dangerous signs before; during; and after childbirth, clean delivery, and first aid in case of an obstetric emergency. Each TBA was paid a symbolic monthly incentive of US$4. Supervisory meetings between TBAs and staff working in health facilities were held monthly. To stimulate demand for institutional childbirth, women delivering in health facilities received baby kits containing a basin, a plastic cup, a bar of soap, and a baby blanket. This study aimed to 1) understand the extent of integration of community-based TBAs in the health system, 2) reveal the factors influencing this integration, and 3) explore the perceived solutions to the challenges community-based TBAs faced in adopting their new roles.

## Materials and methods

### Study setting

This study was conducted in Yirol West County, in the former Lakes State, South Sudan. In 2015, the county had an estimated population of 142,701 people and was divided into 7 *payams* (sub-county administrative units) namely: Abang, Anuol, Geng Geng, Aluakluak, Geer, Mapourdit and Yirol Town. The main ethnic group is Dinka (Atuot, Ciec and Jier clans) and semi-nomadic pastoralism and rudimentary crop farming are the main sources of livelihood for the inhabitants. At the time of the study, Yirol West County was served by two hospitals: Yirol County Hospital (a referral government hospital, which also serves Yirol East and Awerial counties) and St. Immaculate Hospital (a mission hospital in Mapourdit). The county was also served by 8 PHCUs and 2 primary health care centres (PHCCs). In South Sudan, the PHCU is the lowest-level health facility, which is supposed to be staffed by two community health workers (CHWs) and a community midwife while a PHCC is supposed to have one clinical officer, three professionals nurses, two midwives, three CHWs, and lower cadre staff [18]. However, none of the PHCUs and PHCCs in the county had a professional health worker; thus, childbirth services at these facilities were being provided by CHWs and facility-based TBAs. Both hospitals had SBAs. With reference to the TBAs’ pre-integration period (May 2013-April 2014), the number of childbirths after integration (May 2014-April 2015) increased by 13.5% at Yirol Hospital, 26% at St. Immaculate Hospital, and 415% at PHCCs and PHCUs (S1 Table). The coverage of institutional childbirth increased from 25.9% to 36.7% (a 41.7% increase) over the same periods (S1 Table).

### Design and participants

This qualitative study collected data utilising focus group discussions (FGDs) and key informant interviews (KIIs). The FGDs and KIIs were conducted with the aim of exploring the factors affecting the effective integration of TBAs into the health system and the challenges the TBAs were facing. The extent of integration of TBAs in the health system was assessed by inquiring about their current role in provision of maternal health care. Data from KIIs were also used to triangulate the information collected through FGDs.

The study participants consisted of community-based TBAs, facility-based TBAs (TBAs employed by the MoH to work in health facilities), health facility staffs (CHWs in PHCUs/PHCCs and midwives in hospitals), women who delivered in the past one year, village health committee (VHC) members, and staff of the CHD. Administratively, community-based TBAs can be divided into two groups: those who had been recognised by the CHD and efforts had been made to integrate them into the health system (community-based integrated TBAs), and those not recognised by the CHD and thus no efforts had been made to integrate them into the health system (not-integrated TBAs). In the county, there were 185 TBAs recognised by the CHD: 13 working in the hospital; 17 working in PHCUs and PHCCs; and 155 community-based integrated TBAs.

### Sample selection

The number and type of participants to include in the study were guided by our judgement on the extent to which they could contribute towards providing relevant information to respond to the research questions. Thus, we determined the number of KIIs and FGDs *a priori* and used purposive sampling to select participants; ensuring that data are collected from key individuals in different geographical areas.

**Fig 1:**
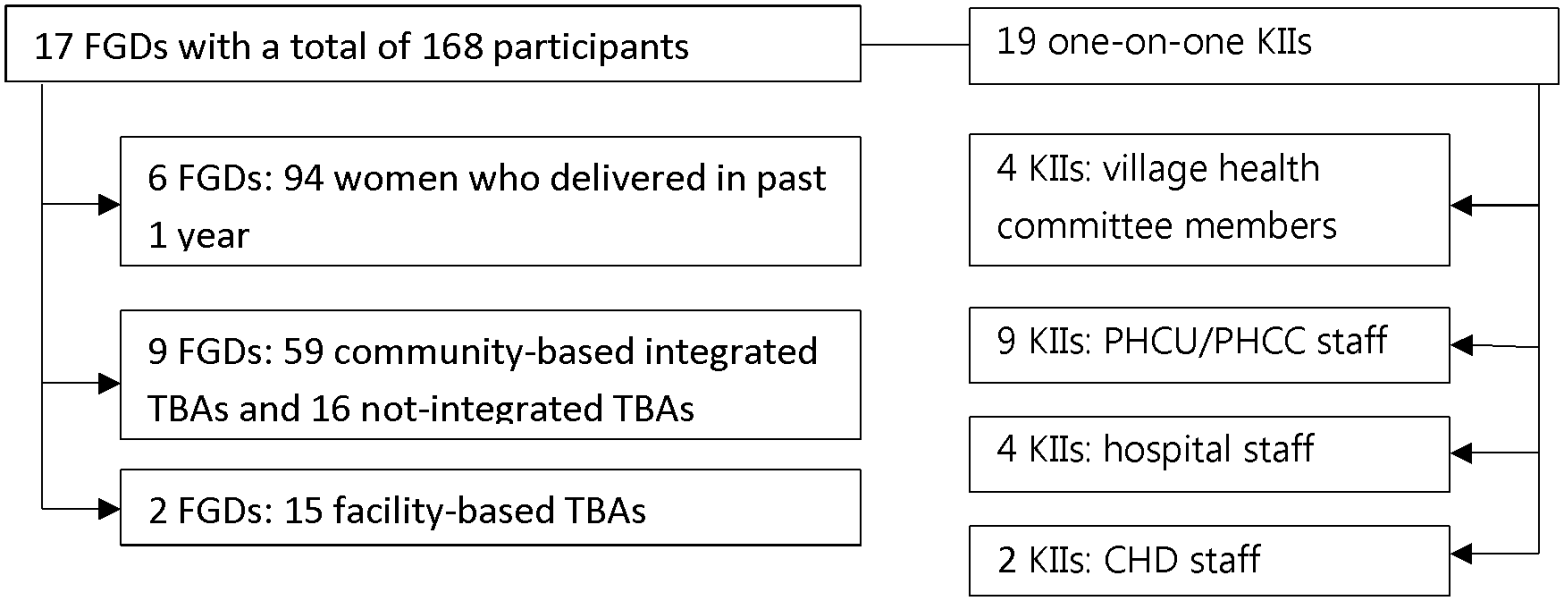
The number of focus group discussions, key informant interviews, and participants. FGD: focus group discussion; KII: key informant interview; PHCC: primary health care centre; PHCU Primary health care unit; TBA: traditional birth attendant.

Data were collected in 5 out of the 7 *payams* mentioned above. Gheer *Payam* was excluded because of insecurity while Yirol *Payam* was excluded because it is close to Yirol Hospital and had a limited TBAs’ activity. Not-integrated TBAs were identified with the help of VHC members, CHWs, and other TBAs. To recruit women FGD participants, one village per *payam* was randomly selected. From each village, women who delivered in the preceding 12 months were purposively selected with the help of VHC members and CHW. The time frame of 12 months was chosen to maximise recall and to ensure the responses were contemporary. A total of 17 FGDs, with an average of 12 participants per group, were conducted (Fig 1). Participants of KIIs were purposively selected amongst individuals judged to have the highest knowledge about maternal health issues in the county based on their job responsibilities, seniority, and working experience. These included: 9 CHWs from 5 PHCUs/PHCCs, 4 hospital maternity staff, 4 VHC members, and 2 CHD staffs (Fig 1). VHC members were from the villages selected for FGDs.

### Data collection

Data were collected in October and November 2015. FGDs and KIIs were conducted utilising pretested open-ended question guides that allowed for flexibility in gathering information. Socio-demographic characteristics of FGD participants were collected using closed-ended questionnaires. Each FGD was conducted by two Dinka (local language) speaking facilitators who were well versed in the local language and culture. One data collector facilitated the sessions while the other one managed audio recordings and took field notes. The data collectors were trained for one day and were supervised by the principal investigator (CW) who was present at all FGDs. The FGDs were conducted in local churches, schools or under trees, as the situation demanded. Each FGD session lasted for about one hour and refreshments were served. Non-participants were not allowed near the FGD venues.

KIIs, each lasting for about 25 minutes, were conducted either in PHCUs (for CHWs and some VHC members), hospitals (for hospital staffs), at the CHD office (for CHD staff), or in villages (for some VHC members). All KIIs, except those with VHC members, were conducted directly in English. KIIs with VHC members were conducted in English with the help of a translator. All FGDs and KIIs were audio-recorded with permission from the participants.

### Data analysis

Audio recordings were transcribed and read through severally to obtain an overall picture of the collected data. The transcripts were then coded by CW based on the content analysis approach [19] using NVivo 10 (QSR International, Melbourne, Australia). After reading through the transcripts, a list of themes was set up in NVivo and was updated as new themes emerged during coding. These themes formed the basis for further data synthesis and interpretation. The themes were summarised and reported under the following sub-headings: 1) New role of TBAs; 2) Facilitating factors in the adoption of the new role by TBAs; 3) Challenges faced by TBAs in adopting their new role; 4) Addressing challenges faced by the TBAs. Segments of text under each theme were then pieced together to provide an overview of the content relating to that specific theme. Quotes were selected to represent a typical response or to illustrate a deviant opinion. A checklist for the Consolidated Criteria for Reporting Qualitative Research (COREQ) [20] is presented in S2 Table.

### Ethical considerations

This study was approved by the ethical committee of the Ministry of Health, Republic of South Sudan. The study was also approved locally by the Yirol West CHD. All study participants were informed about the purpose of the study and what would be expected of them. Participants were also informed of their right to exit the study at any time without any future prejudices. There were no reports of refusal to participate. Because of most participants’ low literacy levels, audio recorded verbal informed consent was sought. This method of obtaining consent was approved by Ethical Committee. Each participant was paid US$4 as a travel cost reimbursement.

## Results

### Characteristics of FGD participants

A majority of the women who participated in the FGDs were aged 20–34 years (75.5%); had no formal education (94.7%); were currently married (93.6%); attended at least one antenatal care visit during their last pregnancy (96.8%); and did not deliver in a health facility (57.4%) as shown in Table 1.

**Table 1:** Characteristics of women who participated in focus group discussions.

Characteristic	Frequency (n = 94)	Percent
Age group		
18–19	9	9.6
20–24	24	25.5
25–29	23	24.5
30–34	24	25.5
>34	10	10.6
Missing data	4	4.3
Parity		
1–2	26	27.7
3–4	28	29.8
5–6	30	31.9
>6	10	10.6
Educational level		
None	89	94.7
Primary	5	5.3
Marital status		
Currently married	88	93.6
Never/formerly married	6	6.4
Attended ANC during last pregnancy		
Yes	91	96.8
No	3	3.2
Delivered in a health facility		
Yes	40	42.6
No	54	57.4

A majority of the TBAs in the FGDs were aged 35–49 years and almost all had no formal education (Table 2). Facility-based TBAs tended to have a longer working experience than community-based TBAs. Some not-integrated TBAs (5/16) were also referring women to health facilities but all of these TBAs were attending to home deliveries. On the other hand, almost all community-based integrated TBAs were referring women to health facilities and slightly less than a third (17/59) were also attending to home deliveries. Most of the not-integrated TBAs (13/16) and some community-based integrated TBAs (13/59) had not received any training organised by the CHD or by CUAMM.

**Table 2:** Characteristics of traditional birth attendants who participated in focus group discussions.

Characteristic	Facility-based TBAs (n = 15)		Community-based integrated TBAs (n = 59)		TBAs not-integrated (n = 16)	
	Frequency	%	Frequency	%	Frequency	%
Age group						
<35	2	13.3	8	13.6	5	31.2
35–49	5	33.3	30	50.8	11	68.8
>49	4	26.7	13	22.0	0	0.0
Missing data	4	26.7	8	13.6	0	0.0
Marital status						
Married	9	60.0	34	57.6	6	37.5
Formerly married	6	40.0	25	42.4	10	62.5
Education						
None	15	100	58	98.3	16	100
Primary	0	0.0	1	1.7	0	0.0
Period working as a TBA						
<10 years	4	26.7	40	67.8	9	56.2
10–19 years	6	40.0	12	20.3	7	43.8
>19 years	5	33.3	7	11.9	0	0.0
Referring women to health facilities						
Yes	—	—	58	98.3	5	31.2
No	—	—	1	1.7	11	68.8
Attending to home births						
Yes	—	—	42	71.2	16	100
No	—	—	17	28.8	0	0.0
Trained by CUAMM/CHD						
Yes	—	—	46	78.0	3	18.8
No	—	—	13	22.0	13	81.2

### The new role of TBAs

There were mixed findings on the activities conducted by the community-based integrated TBAs. In general, it emerged across all FGDs and KIIs that most community-based integrated TBAs were referring and at times, accompanying women to health facilities for childbirth. The TBAs were doing this amidst challenges as will be described later.

‘*TBAs of Panakar [a village in Abang payam] just call the hospital or the PHCU. If the ambulance is not coming, the lady is taken by the TBA up to the hospital or the PHCU*.’

(KII, VHC member, Abang Icholuoth village)

‘*They (TBAs) are helping us by referring mothers in labour to the health facility*.’

(FGD, woman, Angany)

However, it was clear that others, including some community-based integrated TBAs, were still attending to births in the villages and making referrals only when there was a complication. There were some misconceptions about the new role of TBAs even among CHWs.

‘*Our role is to manage labour in the villages and to refer the cases we cannot handle*.’

(FGD, community-based integrated TBAs, Aruau)

‘*If they [TBAs] get a difficult delivery, they refer to the PHCU, but if it is not difficult, they assist*.’

(KII, CHW 2, Mageng PHCU)

There was a general feeling that it was difficult to completely stop community-based TBAs from conducting home deliveries.

‘*That one [changing the role of the TBA] is not working because it is difficult to stop volunteer TBAs [community-based integrated TBAs] from assisting women to deliver at home*.’

(KII, CHW 1, Wou Wou PHCU)

‘*Yes, I cannot deny that [that some TBAs are still conducting home deliveries]. They are, and it has happened several times. TBAs have been told to stop, but some haven’t and we don’t know their interest*.’

(KII, CHD staff 1, Yirol County)

Although some of the TBAs still perceived assisting home deliveries to be part of their role, some were doing this out of necessity, for instance in case it was too late to refer the woman to the health facility or if the health facility was too far. Some TBAs had stopped insisting on home childbirth.

‘*We walk in the whole village to know the number of pregnant mothers and to tell them to go to the health facility. We assist during delivery if the mother is not able to reach the health facility*.’

(FGD, community-based integrated TBA, Anuol)

‘*If they [TBAs] get a woman outside there delivering, they can help her. They also come to report it to the health facility. If she hasn’t delivered at home, they can bring her here [to the health facility]. That is what I have seen them do*.’

(KII, VHC member, Mageng)

Besides referring women to health facilities for childbirth, it emerged from the FGDs and KIIs that community-based integrated TBAs were also referring women for ANC, providing health education on child and maternal nutrition, and referring women perceived to be malnourished or anaemic to health facilities. The activities of community-based integrated TBAs were being monitored through the monthly reports they were submitting to the CHD.

### Facilitating factors in the adoption of new role by TBAs

Facilitating factors in the adoption of the new role by TBAs are summarised in Table 3.

**Table 3:** Facilitators of adoption of new role by traditional birth attendants.

Theme	Main points
Acceptance of the new role of TBAs	Referral of women to health facilities was acceptable to the community, women, and TBAs Women felt safer to be accompanied by TBAs to health facilities TBAs were happy to be contributing towards improving maternal health TBAs were motivated by a feeling of being part of the formal health system
Perceptions about the care in health facilities and the risks of childbirth at home	Institutional childbirth was perceived to be safer than home childbirth Women were satisfied with the care provided in health facilities Women perceived home childbirth by TBAs to be of poorer quality TBAs perceived to be at risk of infection through contact with body fluids during delivery TBAs perceived home delivery to be risky to the mother and her baby TBAs were afraid of facing consequences in case of death or morbidity of the mother and/or baby
Personal commitment and motivation among TBAs	The spirit of serving the community and improving maternal health, with or without incentives among some TBAs
Relationship between community-based integrated TBAs and health facility staff	A good working relationship between community-based integrated TBAs, CHWs in PHCUs and facility-based TBAs CHWs viewed TBAs as co-workers CHWs were involved in training TBAs CHWs helped TBAs in completing monthly reports
The role of baby kits at health facilities	Women delivering in health facility were rewarded with baby kits The kits were synergistic to the work of TBAs
The role of training	Influence of the training from CHD/CUAMM and on-job from CHW and other health workers

#### Acceptance of the new role of TBAs

Generally, the community including women had accepted the new role of TBAs (i.e. the promotion of institutional childbirth). This was mainly because of a gradual realisation that institutional childbirth was safer than home childbirth.

‘*They have accepted [the new role of the TBA]*. *If your wife gives birth in the hospital*, *she* comes *home healthy with a child*. *That is a good thing they have seen*.*’*

(KII, VHC member, Icholuoth village Abang)

‘*Husbands think that the new role is good because the woman and her baby are taken care of by a doctor or somebody who is qualified*.’

(FGD, women, Aruau)

‘*They (women) feel it is safer to deliver in the hospital. They are happy because TBAs are helping them to reach the health facilities*.’

(KII, staff 1, Mapourdit hospital)

Previously, women who wished to deliver in a health facility would face resistance especially from their husbands—this still persists in some cases as will be described later. Additionally, the community used to fear that any woman referred to the hospital would automatically be operated on.

‘*Two years ago, husbands used to refuse to take their wives to the health facility but now after talking to them in churches and in community meetings, they accept*.’

(KII, VHC member, Pabour)

“*When we started the work, some people used to say `I don’t want my wife to deliver here’. But now they are delivering in the health facility. They have calmed down and there is no fighting or complaining*.”

(KII, CHW 2, Mageng PHCU)

While travelling to the health facility, women felt safer to be accompanied by a TBA because the TBA could provide emotional support and help in case of an urgent delivery.

‘*Some women are happy if there is somebody like a TBA to accompany them to the health facility*.’

(FGD, women, Aruau)

Community-based integrated TBAs had a positive attitude towards referring women to health facilities and felt that their new role was contributing towards reducing maternal morbidity and mortality.

‘*We appreciate working for mothers by providing them with a good linkage to the health staffs when we refer them to deliver in the health facility*.’

(FGD, community-based integrated TBA, Anuol)

‘*We like our work because it has stopped maternal deaths, which used to be common in the past*.’

(FGD, community-based integrated TBA, Aruau)

Moreover, the TBAs felt that their new role had elevated their status in the community and were proud to be part of the formal health system.

‘*Our role of accompanying mothers in labour makes us feel like we are part of the government or NGO because we work together and our work is important*.’

(FGD, community-based integrated TBA, Aluak Luak)

#### Perceptions about the care in health facilities and the risks of childbirth at home

Adoption of the new role by TBAs had been expedited by women’s perception that delivering in the health facility was safer than delivering at home. This was related to factors such as the level of hygiene in health facilities, the handling of the baby and the mother, and the availability of light in health facilities (for night time births). Women were also happy with the way they were being received and handled at health facilities following referral by TBAs.

‘*TBAs working in health facilities receive us well, provide good care and are responsible for taking care of us while we are in bed. The care they [facility-based TBAs] provide is better than that provided by the village TBAs*.’

(FGD, woman, Madbar)

On the other hand, some women perceived the care provided at home with the assistance of TBAs to be of poorer quality and unhygienic. Some also had doubts about the competency of community-based TBAs in attending to them during childbirth.

‘*At home, there is no light to see you if it is at night and no razor blade to cut the child’s cord. But in the health facility, all these things are available*.’

(FGD, woman, Aruau)

‘*In the village, blood loss is not controlled and after delivery, very little blood remains in your body*.’

(FGD, woman, Pabour)

TBAs also felt that delivering in health facilities was better than delivering at home. They viewed health facility staffs to be more knowledgeable in handling labour and labour-related complications. They also felt that health facilities had the required infrastructure, equipment, supplies, and drugs for childbirth.

‘*We prefer that women deliver in health facilities because of the heavy bleeding that might come during or after delivery*.’

(FGD, community-based integrated TBA, Panakar)

Moreover, TBAs had realised that assisting women to deliver at home was not only risky to the mother and her baby but also to them (TBAs). They perceived to be at risk of infection through contact with blood during childbirth. It was unclear whether this perception existed prior to TBAs changing their roles.

‘*Infection is a big worry to us because blood may splash on your body while handling a baby*.’

(FGD, community-based integrated TBA, Mageng)

‘*It can be very frightening to the TBA if a mother in labour becomes unconscious. The baby may also be in danger because of lack of air*.’

(FGD, community-based integrated TBA, Panakar)

Additionally, increased awareness of the community about the banning of TBAs from conducting deliveries at home was making referral of women to health facilities to become a norm and TBAs assisting deliveries at home were at risk of a backlash in the case of a complication or death of the mother or baby. Thus, many TBAs had become afraid of taking the risk of conducting home deliveries and were happy to transfer this risk to health facilities.

‘*Some TBAs fear to handle you because if it is outside there and something bad happens to you, she will be held responsible*.’

(FGD, woman, Madbar)

‘*They attempt to assist, but if two hours elapse, they refer the mother or they call the ambulance to transport the mother to the hospital. If the TBA refuses to refer the mother, and the mother gets a problem, the community will blame the TBA and she will face problems*.’

(KII, staff 1, Yirol hospital)

#### Personal commitment and motivation among TBAs

Personal commitment and willingness to help the community by some TBAs were playing a key role in their effective integration in the health system. Some TBAs were highly committed to improving maternal health, with or without incentives.

‘*We are all volunteers and we will still work even without being paid. We can continue working to help our community*.’

(*FGD*, *community-based integrated TBAs*, *Panakar)*

‘*If we inform a TBA that there is a woman here in labour, she [the TBA] will offer herself to help. That TBA will offer herself to come and take the woman to the hospital. Even if it is travelling by foot, she will go with her. That is what I have seen. They are volunteering and they obey because these are their people*.’

(KII, VHC member, Icholuoth village, Abang)

However, this did not apply to all TBAs as will be seen later. Additionally, even self-motivated TBAs were hoping to receive a better incentive one day.

#### Relationship between community-based integrated TBAs and health facility staff

There was a good working relationship between community-based integrated TBAs and CHWs in health facilities. This was partly because both the TBAs and CHWs were from the same localities and knew each other even before the TBAs were instructed to stop attending to home deliveries. Regular meetings between TBAs and CHWs also played a role in fostering the good relationship. Community-based integrated TBAs respected CHWs and viewed them as a source of knowledge. CHWs were involved in training TBAs during the training organised by the CHD and on-the-job. Moreover, CHWs viewed community-based integrated TBAs as colleagues in health service delivery.

‘*We have a good relationship with health professionals; it is like the relationship of a teacher and pupils. The collaboration is good*.’

(FGD, community-based integrated TBA, Agany)

‘*When there is work in the facility, we call them and sit together and do that work… We work together as one group…*.’

(KII, CHW 1, Wou Wou PHCU)

Being illiterate, TBAs relied on CHWs in completing monthly data reporting forms submitted to the CHD; further strengthening the working relationship.

‘*We have a good relationship in connection with monthly reports; those data we collect in the villages on the number of pregnant women and the number of deliveries in the villages*.’

(FGD, community-based integrated TBA, Aluak luak)

Community-based integrated TBAs also had a good working relationship with facility-based TBAs. This working relationship seemed to be important in ensuring a seamless adoption of the new role by community-based integrated TBAs.

‘*We have a good relationship [with facility based TBAs] because we are bound together by work as health providers. All of us face similar challenges even though we have different abilities and skills*.’

(FGD, community-based integrated TBA, Aruau)

#### The role of baby kits at health facilities

The message on the distribution of baby kits to women who deliver in a health facility had spread widely in the community and women were more likely to agree to be referred to health facilities because they knew they would receive this incentive. This incentive was thus synergistic to the work of TBAs.

‘*It is very good to deliver in the health facility because some items such as medicine, soap, basin and other things are given to you and your child*.’

(FGD, woman, Agany)

‘*Women don’t deliver at home because when they come here, they get a lot of things such as soap, basin, and clothes for the child, and the child is vaccinated*.’

(KII, VHC member, Pabour)

#### The role of training

The training (s) that community-based integrated TBAs had received at the start of their integration in the health system seemed to have contributed to the adoption of their new role.

‘*We are happy with the training conducted to improve our work in service delivery to our people, to improve the health of mothers*.’

(FGD, community-based integrated TBA, Mageng)

However, not all community-based integrated TBAs had been trained (Table 2).

### Challenges faced by TBAs in adopting their new role

The challenges faced by TBAs in adopting their new role are summarised in Table 4.

**Table 4:** Challenges faced by traditional birth attendants in adopting their new role.

Theme	Main points
Problems in communicating with health facilities	TBAs lacked mobile phones, money to buy airtime, and means to charge their phones Lack or poor mobile phone network Some TBAs lacked the contact phone numbers of health facilities
Distance to health facilities and transportation problems	Some villages were located in remote villages which hampered referral. TBAs lacked means of transportation and walked to collect data, mobilise communities, and accompany women to health facilities. Ambulance and motorcycles could not access many parts of the county because of poor roads Motorcycles were not preferred by women in labour
Insecurity	TBAs could not escort women to health facilities due to conflicts and fear of wild animals Insecurity affected ambulance and motorcycles movement
Delays in seeking care by women	TBAs attended to home deliveries because women sought help when at an advanced stage of labour
Lack of materials and supplies for TBAs	TBAs lacked basic supplies such as torches, raincoats, gumboots and bags TBAs lacked basic supplies to assist women in case of urgent childbirth
Health system constraints	Lack of qualified staff in PHCUs and PHCCs Infrastructural limitations to handle deliveries at PHCUs and PHCCs
Insufficient monetary incentive/loss of income and other incentives	The monthly incentive of 12 South Sudanese Pounds received by TBAs considered to be too low and did not match the workload and cost of living TBAs were no longer getting the incentives they used to get when assisting home births. The community considered TBAs to be employed and thus was not obliged to give any anything extra to them
Insufficient/lack of training	TBAs not been trained/insufficiently trained. Some TBAs had not understood the rationale for referring women to health facilities
Lack of a common understanding among health staff about the new role of TBAs	Some CHWs thought that the role of community-based integrated TBAs was to attend to deliveries at home and to refer only complicated cases. This can be misleading information to TBAs

#### Problems in communicating with health facilities

TBAs had difficulties in communicating with health facilities whenever they had a woman in labour in need of being transported to a health facility. This was due to many factors including lack of mobile phones and/or means to charge their phones, lack of money to make phone calls, and poor mobile phone network. Additionally, some TBAs did not have the health facility contact phone number. This forced some of them to assist women to deliver at home even if that was not the intention. The problems of communication became more serious at night when movement was restricted by insecurity and lack of lighting.

‘*Poor mobile phone network is a great challenge. Sometimes we are connected to health facilities but on other occasions, even if one of us has a phone and calls the health facility, the call does not go through and this discourages the relatives of the woman*.’

(FGD, not-integrated TBA, Pabour, Madbar)

‘*The challenge is that when a TBA finds a pregnant mother in labour deep in the village at night, communication with the health facility is difficult*.’

(FGD, community-based integrated TBA, Aluak luak)

#### Distance to health facilities and transportation problems

The problem of long distance to health facilities and lack of transportation means was more serious in remote villages not easily accessible by road. Long distance to health facilities meant that there was no time to refer women at an advanced stage in labour or with obstetric emergencies.

‘*Because if it is a faraway village and the woman is about to deliver, there will be no time for her to come. Maybe the lady will deliver there and she will bring only the report*.’

(KII, CHW 1, Mageng PHCU)

Due to poor roads and lack of reliable transportation means, walking was the most common means of getting to health facilities. This posed a great challenge to the work of TBAs in accompanying women in labour to health facilities, collecting data on the number of pregnant women in their localities, and in mobilising communities. Often, TBAs had to walk for long distances to do all these. The available means of transportation (motorcycles at PHCUs and PHCCs and ambulances at the hospitals) could not access many parts of the county because of poor roads. The ambulance at Yirol Hospital was also over-stretched and sometimes delayed to respond to emergency calls from villages. Communication problems, long distances to health facilities and sparsely populated villages exacerbated the transportation problems. Although most PHCUs and PHCCs had motorcycles to pick women in labour from villages, this means of transportation was not preferred by women due to bumpy roads, the fear of accidents, and insecurity. Transportation was harder during the rainy seasons.

‘*If there is a woman who wants to deliver in the village, volunteer TBAs [community-based integrated TBAs] send a child or someone on a bicycle to come and inform the TBA in the health facility. There is no transport. That’s why the number of deliveries is small because there is no transport to reach far places. There is another village with no road to go there and some people don’t even have phones to call*.’

(KII, VHC member, Pabour)

#### Insecurity

The prevailing state of insecurity in Yirol West County affected the work of TBAs. TBAs were afraid to escort women to health facilities especially at night because they feared inter-tribal fighting, rape, and abduction.

‘*There is fear of insecurity due to tribal conflicts, cattle raiding, and the culture of revenge where women are killed even at water points and when collecting firewood*.’

(FGD, community-based integrated TBA, Mageng)

‘*Conflicts don’t allow us to move at night because they rape women of any age; whether young or old*.’

(FGD, community-based integrated TBA, Anuol)

There was also fear of attacks from wild animals especially when moving at night without a light source.

‘*We fear wild animals when moving at night without a torch to light the way to the home of the woman in labour*.’

(FGD, community-based integrated TBA, Anuol)

The insecurity situation also affected the movement of the ambulance and motorcycles used to transport women to health facilities during emergencies.

#### Delays in seeking care

In some instances, TBAs attended to home deliveries because women waited for too long before seeking help and only contacted the TBA when referral was not an option.

‘*….sometimes the mother does not feel ready to come and deliver at the hospital for reasons well known to her. However, she will just call the TBA at the last moment, when the baby is coming out and of course she [the TBA] can’t call [the ambulance] because it would be too late, so she has to remain there and help the mother*.’

(KII, staff 2, Yirol Hospital)

#### Lack of materials and supplies

TBAs lacked basic items such as torches for use at night, raincoats and gumboots for use during the rainy season, and bags to carry their paraphernalia. Some TBAs without any intention of assisting women to deliver at home found themselves doing so because of various barriers including those faced by women in accessing health care (S1 File). For instance, because of long distance, a woman would deliver on the way to the health facility and a TBA would be called into action. TBAs lacked basic supplies to assist women in such situations.

‘*The challenge is a lack of materials such as torches, gumboots, and rain coats*.’

(FGD, community-based integrated TBA, Agany)

‘*We have the challenge of lack of materials such as torches, gloves, razor blades, cotton wool, cord clamps, soap, bags, raincoats, gumboots and containers for carrying water*.’

(FGD, community-based integrated TBA, Aruau)

#### Health system constraints

Although it was the intention of the CHD to have all women deliver in health facilities, in reality the health system in Yirol West was still weak and could not handle all childbirths. Qualified midwives were available only at the two hospitals, and PHCUs/PHCCs were staffed with CHWs and TBAs who had only basic training and skills to attend to childbirth.

“The number of qualified midwives is not adequate, so for the time being, we cannot eliminate them [the TBAs]. And we cannot say: ‘no, you can’t do this’ unless the hospital is equipped and has adequate personnel. Also, the peripheral health facilities have no qualified personnel. They have only this community health workers who are trying their best but are not equipped for deliveries, they are very active in referring.”

(KII, Staff 2, Yirol hospital)

There were also infrastructural limitations at lower level health facilities because they lacked maternity units, sufficient beds, and space to accommodate women in labour, during delivery, and in the postpartum period. Consequently, PHCUs and PHCCs were sometimes struggling to cope with the number of women accessing them (referred by TBAs or not). Some TBAs were aware of this limitation and seemed to be getting discouraged from referring women.

‘*Now, the only problem in my unit is that when the mother delivers, there is no place for her to stay for some minutes or for one hour. When a mother delivers, she is send home because we do not have any place for her to stay. The place is very small*.’

(KII, CHW 1, Panakar PHCU)

‘*Many health facilities don’t have enough beds to accommodate the women in labour whom we refer*.’

(FGD, community-based integrated TBA, Panakar)

Moreover, some women were refusing to be referred to health facilities because they knew about the problem of limited infrastructure.

‘*When labour approaches, some women refuse to deliver in health facilities because there are no beds. There is only one examination coach and there is no bed where mothers can stay for more than 24 hours after delivery*.’

(FGD, facility-based TBA, Yirol West)

#### Insufficient monetary incentive/loss of income and other incentives

Whereas some community-based integrated TBAs had a high degree of personal commitment and self-motivation and were willing to continue working even without a monetary incentive, it emerged in most KIIs and FGDs that the monthly incentive of 12 South Sudanese Pounds (US$4) that the community-based integrated TBAs were receiving was too low to meet the most basic of needs. It was perceived that TBAs were not being compensated fairly for the work they were doing. The nature of their work meant that they could be called upon at any time to accompany women to health facilities. They often spent long hours with women in labour at the expense of working or looking for food for their families. TBAs wondered why the mothers they referred to health facilities were getting a better incentive (baby kits) and their colleagues employed in health facilities were being paid a monthly salary but they were getting almost nothing for their work.

‘*It [the incentive] helps a bit but high prices have affected us and you cannot get anything with 12 South Sudanese Pounds. This little amount of money should be increased…*.’

(FGD, community-based integrated TBA, Aruau)

‘*…they are complaining about salary; there is no work without salary. They are saying that they are in a newly independent country but they have no salary even though they are working. That is why they are not working well because they need a salary. They receive only a motivation, and the motivation is little, just 12 pounds per month*.’

(KII, CHW 1, Mageng PHCU)

Before the TBAs were banned from attending to home deliveries, they would receive mainly non-monetary incentives from the women they assist. But with the change of their role, the TBAs were no longer getting these incentives. Additionally, the community was aware that TBAs were receiving an incentive for referring women and was unwilling to give anything more to TBAs. The community considered TBAs to be employees of CUAMM.

‘*Because of the 12 South Sudanese Pounds, we are no longer being given alcohol, calabash and cups because the community says that we are being paid by the NGO*.’

(FGD, community-based integrated TBA, Anuol)

‘*Nowadays nothing is given to us because the community has heard that we are being paid 12 South Sudanese Pounds*.”

(FGD, community-based integrated TBA, Aruau)

#### Insufficient/lack of training

Key informants opined that some TBAs were still attending to home births either because they had not been trained in the new role or the training received was insufficient. Thus, some TBAs had not understood the rationale for referring women to health facilities. Some TBAs had also not heard about the ban on home births. However, this problem seemed to be applicable mainly to not-integrated TBAs.

‘*Some TBAs have not been told not to help women to deliver at home and they have not been trained*.’

(KII, staff 2, Mapourdit hospital)

‘*It is the way of understanding. Volunteer TBAs [community-based integrated TBAs] understand that all women should be referred to the health facility. But some TBAs who do not understand the MoH directive conduct delivery at home*.’

(KII, CHW 1, Wou Wou PHCU)

#### Lack of a common understanding of the new role of TBAs

It seemed like some formal health care workers were not clear about the new role of TBAs. They thought that the role of community-based integrated TBAs was to attend to childbirth at home and to refer women only if there was a complication. Such health workers are unlikely to support TBAs to stop attending to childbirths at home or they may convey the wrong message to TBAs.

‘*If it is normal [uncomplicated delivery], they can do it there in the community. If there is a difficulty, they can call us and then we can go and pick the woman from the community and bring her to the health facility*.’

(KII, CHW 1, Wou Wou PHCU)

### Addressing the challenges faced by the TBAs

Table 5 presents a summary of the suggested solutions to the challenges faced by the TBAs in adopting their new role.

**Table 5:** Addressing the challenges faced by traditional birth attendants in adopting their new role.

Theme	Main points
Provision of a salary	The current TBAs’ monthly incentive should be increased
Training and supervision	Regular training/refresher training Supervising TBAs to ensure that they are performing their role and to identify and address their challenges
Addressing transport and communication problems	Providing TBAs with bicycles
Improving the health facility infrastructure	Constructing more health facilities Allocating more space for maternity at the existing PHCUs and PHCCs and providing more maternity beds
Providing supplies and equipment to TBAs	Providing basic supplies such as raincoats, gumboots, torches, soap, umbrellas and bags Providing clean delivery kits to TBAs for use in attending to urgent deliveries Providing uniforms to TBAs

#### Provision of a salary

This was the most frequently mentioned solution to keeping TBAs integrated into the health system. This solution emerged at all FGDs and KIIs. Almost all participants opined that the monthly monetary incentive being given to TBAs should be increased to a reasonable amount to match the cost of living and the magnitude of work performed by TBAs.

‘*We need our small incentive to be increased to an amount that can sustain and protect us from the increasing prices of goods on the market*.’

(FGD, community-based integrated TBA, Aluak luak)

‘*I would like you to give TBAs a salary because they are obeying your work. If you go to the house of a TBA at night and tell her that there is a lady here, she will obey and go. That one needs them to have a salary*.’

(KII, VHC member, Icholuoth village Abang)

It was also suggested that TBAs should be given non-monetary incentives instead of a salary.

‘*Instead of money they could be given soap or sugar, this can motivate*.’

(KII, CHW, Anuol PHCC)

#### Training and supervision

Training was perhaps the second most frequently mentioned solution to the challenges faced by the TBAs. The training the TBAs had received was perceived to be inadequate. Moreover, some TBAs had not been trained. Respondents felt that if TBAs were well trained, they would know how to communicate better with women and their husbands in explaining to them the reasons for referral. They would also be able to safely attend to a delivery in case it was not possible to refer the woman to a health facility. It was also noted that the trainings or refresher trainings should be conducted regularly to ensure continuous learning.

‘*Training can be the first priority because many TBAs lack skills*.’

(FGD, community-based integrated TBA, Mageng)

There was also a need for more supervision of TBAs to ensure they were performing their new role and to identify and promptly address their challenges. It was also mentioned that health care workers should continuously communicate with TBAs to encourage them to refer women to health facilities.

‘*They need to be supervised more frequently so that we can know if they are performing, and if they are not performing we can re-organize and bring them here*.’

(KII, staff 2, Yirol hospital)

‘*We should talk to them and understand why they don’t refer women to the health facilities*.’

(KII, CHW 2, Wou Wou PHCU)

#### Addressing transport and communication problems

Participants felt that transport problems could be solved by providing TBAs with bicycles for mobilising and referring women.

‘*If the government or CUAMM partners have no salaries for TBAs*, *they should support TBAs by providing them with bicycles to enable them to work*. *If it is a far away village*, *such as Lual*, *maybe they can come with the bicycle to bring the report*. *If a woman in labour is unable to walk*, *the TBA can come here with the bicycle to inform the PHCU and then we will go with the motorbike to take her*.’

(KII, CHW 1, Mageng PHCU)

There were only a few suggestions to solve the communication problems. This was because even if one had a mobile phone, there would still be challenges such as charging the mobile phones, poor mobile phone network, and buying airtime. Nevertheless, a few TBAs felt that provision of mobile phones could solve the communication problem.

#### Improving the health facility infrastructure

Because of the limited availability of maternity infrastructure to comfortably accommodate women referred to PHCUs and PHCCs, it was proposed that more health facilities should be constructed and more space should be allocated for maternity at the existing PHCUs and PHCCs. Construction of more health facilities was also seen as a way of tackling geographical inaccessibility and the associated transportation problems. Improvement in maternity infrastructure, for example by provision of more beds, was also suggested.

‘*More health facilities should be constructed in the community to ensure that everybody is staying near a health facility for easy access*.’

(FGD, community-based integrated TBA, Wou Wou)

‘*This health facility has only two beds and at night, there might be six, nine or ten women with no bed. They just put children on the floor. It is good to have a separate maternity……’*

(KII, VHC member, Pabour)

#### Providing supplies and equipment to TBAs

It was suggested that community-based integrated TBAs should be given basic supplies pertinent to their new role. Such supplies included raincoats, gumboots, torches, soap, umbrellas and bags for carrying the supplies.

‘*We need to be provided with materials such as torches, gumboots, raincoats, bags, umbrellas and so forth*.’

(FGD, community-based integrated TBA, Anuol)

Because the TBAs found themselves in situations where they needed to help women to deliver, it was suggested that they should also be given clean delivery kits for use while attending to such deliveries.

‘*…. they need the materials to use out there because when the mother starts to deliver in a far place, they try to come but sometimes the clinic is far and the baby is coming out*.’

(KII, CHW 1, Panakar PHCU)

It was also suggested that the TBAs should be given a uniform or T-shirts to make them easily recognisable in the community. The uniform could also contribute towards improving the status of the TBAs in the community.

‘*We can also provide them with uniforms to let them feel that they too are workers*.’

(KII CHW 1, Aruau PHCU)

## Discussion

Our study found that only a bit more than one year after banning TBAs from assisting women to deliver at home in Yirol West, South Sudan, many TBAs were systematically referring women to health facilities for delivery, although some were still attending to deliveries at home, sometimes simply out of necessity. The factors facilitating the acceptance of this new “health promotional” role found in our study are similar to those of studies conducted in Somaliland [15] and Kenya [21]. These studies found that proper training of TBAs, strengthening the capacity of the health system to meet the needs of women, and providing adequate incentives to TBAs easily made the TBAs to change their role. Our findings also largely agree with those of a review of qualitative studies on lay health worker programmes to improve access to maternal and child health [22]. The review found that barriers and facilitators of program implementation were mainly related to programme acceptability, appropriateness and credibility, and health system constraints [22].

Programme recipients generally have a positive attitude towards programmes involving lay health workers [22]. Thus, it is not a surprise that the community and women in Yirol West were positive about the new role of TBAs. Although this can be linked to community mobilisation, incentives for women at health facilities, and positive outcomes (low risk of morbidity and mortality) among women who delivered in health facilities, the trust and respect communities usually have in TBAs seemed to have played a key role. In addition, TBAs are more likely to be self-motivated in helping women, which explains why for some of the TBAs, this was a facilitating factor for their integration in the health system. These reasons probably also underpin the positive attitudes of the TBAs towards their new role as found in this study.

Despite banning TBAs from attending to deliveries at home, some TBAs were nevertheless assisting women delivering at home. Sometimes this was done out of necessity, for example if it was insecure outside or if referral was not an option because the woman delayed to seek care. In line with our study, some studies have reported that women avoided going to the health facility by contacting the TBA when it is too late to refer [23, 24]. In conflict-ridden settings, insecurity can be a major barrier to service delivery and access [25]. Indeed, the existing fragile state of insecurity in South Sudan was restricting access to health facilities in the country [26]. Apart from human conflict, this study found that fear of attacks from wild animals was affecting the referral of women by TBAs. This fear forced TBAs to choose home delivery over braving a dangerous journey to the health facility at night.

Training, supervision and incentives have been reported as key strategies in changing the role of TBAs to become institutional delivery promoters [11, 15, 27]. It is thus not surprising that these issues emerged as challenges of integrating TBAs in the health system. Traditionally, in Yirol, as is in other parts of South Sudan, TBAs would receive monetary and non-monetary incentives when they assist women to deliver at home. Thus, their integration into the health system without sufficient compensation meant loss of a source of livelihood. In a study in Somaliland, TBAs were not happy after the incentives they were receiving ceased following the end of the project that was supporting them. This demonstrates the importance of ensuring a stable source of income to sustain the integration of TBAs in the health system. Although some TBAs were self-motivated and willing to continue to work even without an incentive, it is uncertain whether this is sustainable in the long term. Moreover, asking TBAs to abandon their traditional trade and source of income and expecting them to work as volunteers, sometimes over long hours and in a difficult and insecure environment, without a reasonable compensation raises ethical issues. Alternatively, WHO task-shifting recommendations have suggested interventions for maternal and newborn health that can be safely shifted to lay health workers if necessary to optimise health workers’ roles. Such task-shifting strategy would expand the role of the TBAs in South Sudan and is likely to improve access to care and foster the integration of TBAs in the health system [16].

Although training of TBAs is important in integrating them in the health system as highlighted in this study, this alone may not be effective if the TBAs are not supervised [27]. In the present study, although some health facility staff were meeting with TBAs on a monthly basis, the health facility staff were unable not solve the fundamental challenges faced by TBAs. Thus, there was also need for frequent meetings between the TBAs and the CHD to promptly identify and troubleshoot the TBAs’ challenges.

TBAs are ubiquitous in South Sudan and this is the first study on their integration in the health system. This study collected data from diverse sources including TBAs, CHWs, professional health workers and managers, VHC members and women. This, together with the triangulation of the data from the different sources ensured that the main issues and perceptions about changing the role of TBAs were captured. However, the study has some limitations. FGD facilitators were generally unskilled in qualitative research and required more training than could be provided given the available time and resources. Secondly, it is possible that some information was lost in translation and transcription. We minimised this by frequently providing feedback on the quality of the translation and clarifying unclear statements with help of the translators. Third, we excluded one *payam* because of insecurity. Although insecurity is likely to have a greater influence on the integration of TBAs in the excluded *payam*, the exclusion of this *payam* is unlikely to have affected our findings and conclusions. Finally, saturation of information should be the basis for determining the sample size in qualitative studies. However, for planning purposes and given the available resources, we determined *a priori* the number of KIIs and FGDs to conduct. To minimise the risk of not achieving saturation because of this approach, we decided to include a higher than the minimum number of subjects required to reach saturation. A number of studies have attempted to determine the required sample size to reach saturation in qualitative studies. For FGDs, the number ranges from 3 to 8 groups [28–30] whereas for interviews, the range is 9 to 16 [30–32]. Thus, our sample of 19 participants for KIIs and 17 FGDs was probably more than what was required to reach saturation. Although we did not formally check for saturation, we noted that the latter interviews and FGDs were yielding no more new information. Therefore, it is unlikely that our results and hence our conclusions would have changed by including more participants into the study.

Overall, the findings of this study suggest that there is a great opportunity to effectively integrate TBAs in the formal health system in Yirol West County through leveraging the factors that facilitate the adoption of new role by TBAs and by tackling the challenges they face in assuming their new role, including addressing the existing barriers to maternal health service access in the county. In addition to the recommendations made by the respondents, formal health workers need to be re-oriented on the new role of community-based integrated TBAs. This is so as to clear misconceptions and improve the supervision of the TBAs. The issuance of baby kits in health facilities should continue because it is synergistic to the work of the TBAs. To partly solve the problem of communication, the CHD should consider introducing toll-free health facility phone numbers. Similar studies in other parts of the country are warranted.

## Supporting information

S1 Table Number of institutional deliveries in Yirol West County before and after integration of traditional birth attendants in the health system. CHW, Community health Worker; PHCC, Primary health care centre; PHCU, primary health care unit; TBA, traditional birth attendant, SBA, skilled birth attendant.
(DOCX)

S2 Table Consolidated criteria for reporting qualitative studies: 32-item checklist S1 File. Barriers faced by women in accessing institutional delivery care in Yirol West County, South Sudan.
(DOCX)

S1 File Barriers faced by women in accessing institutional delivery care in Yirol West County, South Sudan. (DOCX)

S2 File Supporting information file 2. (SAV)

S3 File Supporting information file 3. (SAV)
