# Supplementary material for: Changing the role of traditional birth attendants in Yirol West County, South Sudan
Source: PLoS One. 2017 Nov 2;12(11):e0185726. doi: 10.1371/journal.pone.0185726 (PMC5667815; doi:10.1371/journal.pone.0185726)
Supplement: S1 Table — CHW, Community health Worker; PHCC, Primary health care centre; PHCU, primary health care unit; TBA, traditional birth attendant, SBA, skilled birth attendant. (DOCX) [file pone.0185726.s001.docx]

| Indicator | | May 2013 to April 2014 | May 2014 to April 2015 | % increase |
| --- | --- | --- | --- | --- |
| Yirol County Hospital | |  |  |  |
|  | Number of deliveries assisted by SBAs | 1,262 | 1,432 | 13.5 |
|  | Number of deliveries assisted by facility-based TBAs | 0 | 0 | 0.0 |
|  | Number of institutional deliveries | 1,262 | 1,432 | 13.5 |
| St. Immaculate Hospital | |  |  |  |
|  | Number of deliveries assisted by SBAs | 190 | 240 | 26.3 |
|  | Number of deliveries assisted by facility-based TBAs | 0 | 0 | 0.0 |
|  | Number of institutional deliveries | 190 | 240 | 26.3 |
| PHCCs and PHCUs | |  |  |  |
|  | Number of deliveries assisted by SBAs | 0 | 0 | 0.0 |
|  | Number of deliveries assisted by CHWs and facility-based TBAs | 120 | 618 | 415.0 |
|  | Number of institutional deliveries | 120 | 618 | 415.0 |
| Yirol West County | |  |  |  |
|  | Number of deliveries assisted by SBAs | 1,452 | 1,672 | 15.2 |
|  | Number of deliveries assisted by facility-based TBAs | 120 | 618 | 415.0 |
|  | Number of institutional deliveries | 1,572 | 2,290 | 45.7 |
|  | Number of expected of deliveries | 6,060 | 6,244 |  |
|  | Coverage of institutional delivery (%) | 25.9 | 36.7 | 41.7 |
