# Supplementary material for: Changing the role of traditional birth attendants in Yirol West County, South Sudan
Source: PLoS One. 2017 Nov 2;12(11):e0185726. doi: 10.1371/journal.pone.0185726 (PMC5667815; doi:10.1371/journal.pone.0185726)
Supplement: S2 Table — Barriers faced by women in accessing institutional delivery care in Yirol West County, South Sudan. (DOCX) [file pone.0185726.s002.docx]

| No. Item | Guide questions/description | Reported on page |
| --- | --- | --- |
| **Domain 1: Research team and reflexivity** |  |  |
| *Personal Characteristics* |  |  |
| 1. Interviewer/facilitator | Which author/s conducted the interview or focus group? | Page 9 |
| 2. Credentials | What were the researcher’s credentials? E.g. PhD, MD | The journal does not require this to be specified. The credentials of the researchers are as follows;  CW: MPH, MSc; GDO: MD; CS: MSc; GP: MD; BWL: MPH, PhD, RT: MPH, FM: MD; GS: PhD; and APB: PhD |
| 3. Occupation | What was their occupation at the time of the study? | Page 1 provides information on affiliations.  CW was a doctoral candidate, GDO as the project manager, CS was the country manager, GP and GS were operational research staff, RT was an Assistant Professor, and APB was a Medical Officer |
| 4. Gender | Was the researcher male or female? | CS, GS and APB are all female. All the other authors are male. |
| 5. Experience and training | What experience or training did the researcher have? | CW and APB have experience in conducting qualitative studies on maternal health |
| **Relationship with participants** |  |  |
| 6. Relationship established | Was a relationship established prior to study commencement? | There was no prior relationship between researchers and FGD facilitators and participants. |
| 7. Participant knowledge of the interviewer | What did the participants know about the  researcher? e.g. personal goals, reasons  for doing the research | This information was provided during data collection (page 9) |
| 8. Interviewer characteristics | What characteristics were reported about the inter viewer/facilitator? e.g. Bias, assumptions, reasons and interests in the research topic | Page 9 |
| **Domain 2: study design** |  |  |
| *Theoretical framework* |  |  |
| 9. Methodological orientation and Theory | What methodological orientation was stated to underpin the study? e.g. grounded theory, discourse analysis, ethnography, phenomenology, content  analysis | Page 9 |
| *Participant selection* |  |  |
| 10. Sampling | How were participants selected? e.g. purposive, convenience, consecutive, snowball | Pages 8 |
| 11. Method of approach | How were participants approached? e.g. face-to-face, telephone, mail, email | Pages 8 |
| 12. Sample size | How many participants were in the study? | Page 8 |
| 13. Non-participation | How many people refused to participate or dropped out? Reasons? | Page 10 |
| *Setting* |  |  |
| 14. Setting of data collection | Where was the data collected? e.g. home, clinic, workplace | Pages 6 |
| 15. Presence of non-participants | Was anyone else present besides the participants and researchers? | Page 9 |
| 16. Description of sample | What are the important characteristics of the sample? e.g. demographic data, date | Pages 7, 10-12 |
| *Data collection* |  |  |
| 17. Interview guide | Were questions, prompts, guides provided by the authors? Was it pilot tested? | Page 9 |
| 18. Repeat interviews | Were repeat interviews carried out? If yes, how many? | No |
| 19. Audio/visual recording | Did the research use audio or visual recording to collect the data? | Pages 9 |
| 20. Field notes | Were field notes made during and/or after the interview or focus group? | Page 9 |
| 21. Duration | What was the duration of the interviews or focus group? | Page 9 |
| 22. Data saturation | Was data saturation discussed? | Page 38 |
| 23. Transcripts returned | Were transcripts returned to participants for comment and/or correction? | No |
| **Domain 3: analysis and**  **findings** |  |  |
| *Data analysis* |  |  |
| 24. Number of data coders | How many data coders coded the data? | Page 9/10 |
| 25. Description of the  coding tree | Did authors provide a description of the coding tree? | No. Intermediate documentation is available upon request. |
| 26. Derivation of themes | Were themes identified in advance or derived from the data? | Page 9/10 |
| 27. Software | What software, if applicable, was used to manage the data? | Page 9 |
| 28. Participant checking | Did participants provide feedback on the findings? | No |
| *Reporting* |  |  |
| 29. Quotations presented | Were participant quotations presented to illustrate the themes/findings? Was each quotation identified? e.g. participant  number | Pages 13-35. |
| 30. Data and findings consistent | Was there consistency between the data presented and the findings? | Yes |
| 31. Clarity of major themes | Were major themes clearly presented in the findings? | Yes |
| 32. Clarity of minor themes | Is there a description of diverse cases or discussion of minor themes? | Only major themes and sub-themes were presented |
