# Supplementary material for: Changing the role of traditional birth attendants in Yirol West County, South Sudan
Source: PLoS One. 2017 Nov 2;12(11):e0185726. doi: 10.1371/journal.pone.0185726 (PMC5667815; doi:10.1371/journal.pone.0185726)
Supplement: S1 File — (DOCX) [file pone.0185726.s003.docx]

Women faced various barriers in accessing institutional delivery in Yirol West County. Although some of these factors such as distance were also affecting TBAs directly, they were acting at the level of a woman to reduce the effectiveness of the new role of TBAs. The common barriers in the county were: domestic chores including care of children at home, cultural factors, long distance to health facilities, fear of operation, lack of birth preparedness, and the presence of male birth attendants/lack of privacy in health units as described below.

1. ***Care of children at home and other domestic chores***

Usually, women in Yirol are responsible for performing household chores such as taking care of children, looking for and preparing food, ensuring cleanliness of the house, among others. Such chores prevent women from being admitted to health facilities for delivery if there is no responsible person to leave at home.

*“Another reason is that, if the facility is far away, the mother who is to deliver will find it hard to provide food to children because she is the one who usually stays at home with children while the husband is in the cattle camp.”* (FGD, integrated TBA, Aluak luak)

*“Others fail to go to the health facility because they don’t have a responsible person at home and there is nobody to remain with children because the husband is not able to cook.”* (FGD, women, Lual Itong)

1. ***Cultural factors***

Although the community in Yirol West had changed its perceptions about institutional delivery, there were still pockets of resistance towards institutional delivery, especially among men. Some husbands were still insisting on home birth because of their strong cultural beliefs.

*“If you deliver in the health facility, the husband starts complaining saying that ‘we were born by our mothers those days without these health facilities.’….. This is the main reason why we don’t reach to the health facility quickly.”* (FGD, women Lual Itong)

*“Even one man told us that he will never allow his wife to deliver in the hospital, we asked him why. He said: ‘the reason is I want her to deliver in my presence at home and she must mention how many men she has ever gone with.* *Sometimes here, if the labour goes a bit longer, the mother is asked if she has other boyfriends or another man, to mention so that the baby may come out.’”* (KII, staff 2, Yirol hospital)

1. ***Long distance to health facilities***

This barrier affected both TBAs and women being referred. Long distance to health facilities combined with poor roads and lack of reliable means of transportation forced many women to deliver at home.

*“If there is no transport to take you there then you can be assisted by a TBA at home*.” (FGD, women, Madbar)

*“The only complaint I have received from the community is distance. Like now, we have a place called Aleleng’ chok, if you go footing, it takes four hours. They come to this unit all the way from that place. There is no any other unit. For pregnant women trying to come to the unit from that place, the problem is distance.”* (KII, CHW 2, Aruau PHCU)

Long distance to health facilities was sometimes exacerbated by the semi-nomadic lifestyle of the population in Yirol, and was a risk factor for adverse maternal outcomes.

*“Some of these mothers live very far, to be honest, and they are also mobile; for example the ones who live mostly in the cattle camps. They move from one place to another, they are not stable and they prefer to stay in the cattle camp because they are comfortable in that manner.”* (KII, staff 2, Yirol hospital)

*“This child I am carrying here is not mine but is an orphan of my sister who during pregnancy was living full-time in the cattle camp without attending the clinic. She delivered on her way to the health unit and was taken to the doctor but she was already weak because of delivering and passed away.”* (FGD, women, Agany)

1. ***Fear of operation***

Some women had a misconception that women referred to health units are usually operated on. This might have been because in the past, only women with complications such as obstructed labour would be referred to the hospital and such cases were more likely to deliver through caesarian section. Women wanted to avoid delivering by caesarian section because they felt that they would be limited in the number of children they can have.

*“Most of them are afraid of operation if there is an obstructed delivery. They say the woman cannot deliver vaginally again if she has been operated on three times. That is why most of them refuse to allow their wives to deliver in the unit.”* (FGD, integrated TBA, Aluak luak)

1. ***Lack of birth preparedness***

Lack of basic birth preparedness was cited as a reason why many women were still delivering in the villages. Due to lack of preparedness, women could deliver anywhere including along the road, at the water pump, or on the farm. Thus, TBAs would be called in to help when it was already too late to refer.

*If you have conceived, you prepare yourself and wait for your delivery. But some women don’t do this.”* (FGD, women, Lual Itong)

*“Some women don’t know their expected day of delivery and so they are not well prepared.”* (KII, staff 1, Mapourdit hospital)

Due to lack of preparedness, some women said that the reason they did not deliver in a health facility was because of sudden labour.

*“Labour can begin suddenly and the only option is to deliver at home.”* (FGD, women, Kadula)

“*Labour can start anywhere because a majority don’t know their last menstrual day that would help in knowing their expected day of labour.”* (FGD, volunteer TBA, Lual Itong)

1. ***Male birth attendants/lack of privacy***

Some women did not want to deliver in health units because they were concerned about lack of privacy and the presence of male attendants during delivery in these units.

*“Your belly is turned up and they expose your vagina for all the people to see you.”* (FGD, women, Madbar)

*“They are afraid about the hospital because some staffs are male. So mothers are afraid because they are going to be seen naked by a man. The other is about privacy, it is so important to the mother to be examined or to deliver in a private place where she is not going to be seen, where there is only you and her.”* (KII, CHD staff 2, Yirol West)
